# 3D bioprinting of stem cell-laden cardiac patch: A promising alternative for myocardial repair

**DOI:** 10.1063/5.0030353

**Published:** 2021-07-27

**Authors:** Sanskrita Das, Hyoryung Nam, Jinah Jang

**Affiliations:** 1 Department of Convergence IT Engineering, POSTECH, 77 Cheongam-ro, Namgu, Pohang, Kyungbuk 37673, Republic of Korea; 2 Department of Mechanical Engineering, POSTECH, 77 Cheongam-ro, Namgu, Pohang, Kyungbuk 37673, Republic of Korea; 3 School of Interdisciplinary Bioscience and Bioengineering, POSTECH, 77 Cheongam-ro, Namgu, Pohang, Kyungbuk 37673, Republic of Korea; 4 Institute for Convergence Research and Education in Advanced Technology, Yonsei University, 50 Yonsei-ro, Seodaemun-gu, Seoul 03722, Republic of Korea

## Abstract

Stem cell-laden three-dimensional (3D) bioprinted cardiac patches offer an alternative and promising therapeutic and regenerative approach for ischemic cardiomyopathy by reversing scar formation and promoting myocardial regeneration. Numerous studies have reported using either multipotent or pluripotent stem cells or their combination for 3D bioprinting of a cardiac patch with the sole aim of restoring cardiac function by faithfully rejuvenating the cardiomyocytes and associated vasculatures that are lost to myocardial infarction. While many studies have demonstrated success in mimicking cardiomyocytes' behavior, improving cardiac function and providing new hope for regenerating heart post-myocardial infarction, some others have reported contradicting data in apparent ways. Nonetheless, all investigators in the field are speed racing toward determining a potential strategy to effectively treat losses due to myocardial infarction. This review discusses various types of candidate stem cells that possess cardiac regenerative potential, elucidating their applications and limitations. We also brief the challenges of and an update on the implementation of the state-of-the-art 3D bioprinting approach to fabricate cardiac patches and highlight different strategies to implement vascularization and augment cardiac functional properties with respect to electrophysiological similarities to native tissue.

## I. INTRODUCTION

Cardiovascular diseases (CVDs), which are well recognized as a non-communicable killer disease, account for 32% of the global deaths and comprise a broad terminology, assigned for a set of pathologies that affect the heart.^1,2^ Myocardial infarction (MI), generally recognized as a “heart attack,” occurs due to partial or complete obstruction of one of the coronary arteries, which irrevocably decreases or stops blood supply to the heart and causes necrosis of the cardiac tissue.^3^ Billions of cells, reaching up to 25% of the total left ventricular mass, are lost within few hours post-MI.^1^ Eventually, scar tissue is formed in the injured or necrotic cardiac muscle, which is incapable of conducting electrical or mechanical stimuli and leads to abnormal contractility of the heart.^4^ Approximately 25% of MI patients tend to have left ventricular dysfunction (LVD) and continue to be at a high risk of progressive heart remodeling.^5^ In addition, 36% of MI survivors are expected to develop the risk of heart failure.^6^ With an increasing number of patients and extremely expensive treatment, MI represents a significant monetary burden to the healthcare systems.^1^ The limitation in treatments is due to the previously prevailing viewpoint of the heart being terminally differentiated and incapable of regeneration endogenously in the post-natal life.^7,8^ However, it is now understood that the heart has the very restricted ability to regenerate cardiomyocytes by itself (approximately 1% of cardiomyocytes turnover per year in adulthood).^9^ With documented findings, it has been reported that at the age of 20, the cardiomyocytes' renewal rate reaches 1%, and at the age of 70, it reduces to 0.4% per year.^1^

Existing pharmacological approaches with drugs (e.g., β-blockers, thrombolytic agents, angiotensin-converting enzyme inhibitors, neprilysin inhibitors, and aldosterone antagonists) are often considered as the first treatment options offered to the patients.^10,11^ However, they provide only a temporary solution, rather than effectively rejuvenating the diseased myocardium.^12^ Other strategies, such as invasive balloon angioplasty, stent insertion, and invasive coronary artery bypass grafting, can only slow or retard the advancement to end-stage heart failure and extend human life.^10^ These surgical approaches still face the challenge to either avert or reverse disease progression, thereby causing a continuous surge in hospitalization and mortality rates.^13,14^ To date, “heart transplantation” remains the gold standard, life-extending, curative, and the definitive option to improve cardiac function in patients with end-stage heart failure.^10,13^ Nevertheless, serious concerns associated with heart transplantation, including (1) long waiting time, (2) insufficient organ availability, (3) immune rejection, and (4) high incidences of post-operative complications, with patients depending on lifelong immunosuppressive regimen, can significantly impact the recipient's quality of life.^4,8,15^ Thus, it is recognized that heart transplantation is a non-pragmatic and difficult option for most patients. Similarly, medical practices involving autologous or allogeneic tissue grafting or synthetically made constructs are associated with challenges pertaining to immune rejection, anticoagulation therapy, and limited durability.^16,17^ Therefore, the development of a new, advanced, and cost-effective modality that can significantly benefit heart failure patients in terms of (1) reducing adverse cardiac remodeling, (2) promoting stable repair or regeneration of myocardial functions that are lost to an ischemic event, and (3) correcting the molecular or genetic defects that are cues to disease o nset and its progression is urgently required.

An ideal approach to regenerate a damaged or diseased myocardium is to either promote the proliferation of resident cardiomyocytes *in vivo* or exogenously provide new cardiomyocytes to replace the necrotic tissue.^14^ Cell-based therapy or cellular cardiomyoplasty, in which cells are injected into the infarcted myocardium, has gained attention as a potential and highly promising treatment strategy that aims to prevent or reverse injured myocardial function and promote endogenous cardiac tissue repair or regeneration in MI survivors.^10,15,18^ Preclinical and clinical studies have demonstrated that cell-based therapies attenuate myocardial damage.^8^ Although the detailed mechanisms are insufficiently addressed, it is believed that paracrine signaling essentially takes part in cell-mediated tissue repair.^19–21^ Nevertheless, there are several caveats to cell-based therapies, and their clinical impact is severely hampered by (1) a low rate of cell retention or engraftment after delivery,^22^ (2) a low viability of injected cells,^15^ (3) an insufficient oxygen and nutrient supply, and (4) a lack of proper integration with the recipient's tissue.^23^ Moreover, with continuous attempts to identify the optimal cell types, interest has also shifted toward adult or pluripotent stem cells.

Bioengineering approaches have been developed as alternative treatment strategies to increase the efficacy of transplanted cells by enhancing their retention or engraftment during the early integration phase.^13^ In particular, cardiac tissue engineering endeavors to mimic the native microenvironment of the host cardiac tissue using biocompatible materials loaded with living cells, bioactive molecules, and various stimulating factors.^15,16^ The most widely applicable biocompatible material is a biodegradable polymer, especially for the construction of 3D-structured tissues and organs. However, the synthetic biodegradable polymers are usually rigid to support the dynamic motion of cardiovascular tissues and difficult to be remodeled by the cells, resulting in a gradual decrease in the number of intercellular connections. It can also cause inflammatory reactions and pathological fibrotic states by polymer degradation.^24^ To overcome this, cell sheet engineering was developed to create a 3D structure by separating and layering 2D monolayers of cells that are grown at confluence without biodegradable polymers.^14,25^ The functional and thick engineered cardiac patch encompassing living cardiomyocytes is fabricated by layering the detached cell sheets and is used to replace or repair the damaged myocardium.^14^ Ishigami *et al.* demonstrated functional recovery of the infarcted myocardium in a porcine MI model using clinical-sized large cardiac tissue sheets. The sheets consisted of various human-induced pluripotent stem cells (iPSCs)-derived cardiac cells, including cardiomyocytes, vascular endothelial cells (ECs), and vascular mural cells.^26^ Although a cell sheet-based bioengineered cardiac patch represents an effective tool to study tissue morphogenesis and its functionalities,^4^ such as the intrinsic limitations to recreate biological properties, its functionality, microenvironment, dynamic interactions with niche cells, and signaling pathways similar to the native complexity of human tissues restrict their applications.^27^ In addition, uncertainties associated with the number of cell layers that can be stacked to avoid interruptions in nutritional supplies to the cells create serious hindrance to the effective utilization of the cell sheet-based cardiac patch. Addressing these limitations, 3D bioprinting serves as a potential and promising modality to develop a functional cardiac tissue patch in a geometrically precise and organized manner to replace or regenerate a diseased or damaged myocardial tissue.^4,28^ Hence, with the aim of restoring the functions of a damaged myocardium, efforts have been made to fabricate the 3D bioprinted cardiac patch involving either natural (e.g., alginate,^29^ gelatin,^30,31^ and fibrin^4^) or synthetic hydrogels or decellularized extracellular matrix-based bioinks (dECM)^32^ encompassing living stem cells or their derivatives either singly or in combination with others.

This review discusses the types of candidate stem cells with cardiac regenerative potential (e.g., adult and pluripotent stem cells) and elucidates their applications and limitations. Furthermore, we provide challenges and an update on the implementation of the state-of-the-art 3D bioprinting approach to fabricate the cardiac patch based on the use of either types of stem cells or a combination of cells. In addition, we highlight the different approaches to implement vascularization and augment cardiac functional properties with respect to electrophysiological similarities to the native tissue, which can further aid in myocardial repair.

## II. CANDIDATE STEM CELLS IN MYOCARDIAL REPAIR

Interest in stem cell-mediated cardiac repair has significantly increased with accumulating evidence from preclinical and clinical studies. Stem cells possess properties such as plastic adherence, self-renewal, immunomodulation, and multilineage transdifferentiation.^33–35^ These cells also release growth factors and extracellular vesicles that assist in cardiac repair by paracrine effects. Acellular-based approaches that maximize these effects are also being actively researched for cardiac regeneration.^36–38^ Multiple types of stem cells from autologous or allogeneic sources are being exploited for cardiac regeneration. Broadly, they can be categorized into (1) multipotent adult stem cells [e.g., bone marrow (BM)-derived mesenchymal stem cells, skeletal myoblast, and cardiac stem cells (CSCs)] and (2) pluripotent stem cells (e.g., embryonic and induced pluripotent stem cells), wherein either the differentiated cells are used for transplantation or differentiated *in situ* post-transplantation.^1,12^

### A. Multipotent adult stem cells

Multipotent adult stem cells can be generated from different sources (e.g., BM, peripheral blood, or resident tissues). Because adult stem cells possess cardiogenic differentiation capability, the focus is now shifting toward the development of stem cell sources for repairing or regenerating the damaged myocardial tissues.

Mesenchymal stem or stromal cells (MSCs) are characterized by their (1) self-renewal ability, (2) low immunogenicity, (3) multipotency to transdifferentiate, (4) plastic adherence characteristics, (5) homing ability to tissue injured areas, and (6) participation in vasculogenesis and myogenesis.^8,33,39,40^ MSCs are a rich source of growth factors that substantially contribute to cardiac repair through the proangiogenic, anti-apoptosis, anti-inflammatory, and fibrosis by secreting secretomes.^7,30,41^ In practice, MSCs infused into the MI mouse model are activated to secrete anti-inflammatory proteins in the lung, thereby improving the myocardial infarction and increasing cardiac function.^42^ MSCs are the majorly used cells in preclinical research as well as relevant clinical settings for cell-based therapy.^8,10^ MSCs are harvested from the BM, skin, muscle, and adipose tissue, with BM aspirates and adipose tissue being the common sources for experimental use.^8,39^ Importantly, MSCs are immune-privileged or possess potent immunosuppressive properties, in that they express low levels of major histocompatibility complex (MHC) class I and also lack MHC class II and B-7 costimulatory molecule expressions, making them excellently suitable for use as allografts.^7,10,39^ In experimental systems, MSCs are differentiated into cardiomyocytes and ECs *in vivo* upon transplantation to the myocardium in both non-injury and MI models. When characterized with immunohistochemistry, differentiated MSCs show specificity for both cardiac and ECs and gap junction proteins.^43,44^ Despite low engraftment rates of MSCs, a numerous *in vivo* studies have reported improvements in cardiac function.^45,46^ However, in one such study, electrophysiological analysis revealed that the differentiated cardiomyocytes did not demonstrate electrical properties comparable to those obtained from native cardiomyocytes.^47^ Similarly, in terms of clinical studies, many trials have been conducted to examine the therapeutic potency of MSCs (either with autologous or allogeneic sources) to regenerate a diseased or damaged myocardium.^48,49^

Another cell source selected for cardiac regeneration is BM-derived mononuclear cells (BM-MNCs), because these cells are widely available and also feasible to isolate from patients through BM aspiration.^50^ However, several clinical trials have reported significant but marginal improvement in the resulting cardiac function with BM-MNCs.^10^ Perin *et al.* transplanted autologous BM-MNC in patients with coronary artery disease or LV dysfunction and found no differences in any of the outcomes, including cell therapy and clinical improvement between placebo and BM-MNCs.^51^ Similarly, skeletal myoblasts are used for cardiac cell therapy and were one of the first cell types to be evaluated.^52^ In this regard, Shudo *et al.* demonstrated improved therapeutic benefits of primary skeletal myoblasts in a rat MI model by co-culturing the myoblasts with MSCs; the addition of MSCs to skeletal myoblasts' cell sheets led to enhanced angiogenesis due to increased secretion of a hepatocyte growth factor (HGF) and a vascular endothelial growth factor and consequently augmented cardiac functional recovery in the MI model, thereby suggesting it as a strategy for clinical applications.^53^

Similar to MSCs, resident CSCs are multipotent and self-renewing, differentiate *in vitro*, and secrete cytokines and growth factors that stimulate endogenous stem cells and regeneration mechanisms through a strong paracrine signaling.^7,10,54,55^ In humans, autologous CSCs are derived from either surgical or endomyocardial biopsies and are expanded clonally *in vitro*.^56^ CSCs are majorly found in the atrium and ventricular apex of the heart, albeit at a very low density of one cell per 10 000 cardiomyocytes.^54^ The first reported primitive CSCs were isolated and identified based on the expression of CD117 or c-kit.^10^ In addition, CSCs are positive for stem cell antigen-1, MDR-1 (ABCG2), and markers specific to cardiac stem and progenitor cells (e.g., CD166, PDGFrα, CD105, and CD90).^10^ Beltrami *et al.* successfully isolated c-kit^+^ CSCs from a rat heart and showed their multipotency *in vitro* and the ability to regenerate cardiomyocytes and blood vessels following MI.^54^ To further evaluate the safety of CSCs, a study was performed wherein 20 × 10^6^ c-kit^+^ CSCs were infused into swine hearts via the intracoronary route.^57^ The results demonstrated neither liver nor renal damage nor any myocardial injury due to microembolism. Nevertheless, cellular retention in the myocardium remained low despite the infusion of high cell numbers.

Although the risks in terms of immunogenicity and possible arrhythmic occurrence related to adult stem cell transplantation are usually rare and safety and feasibility have been proven in various preclinical and clinical studies, the cardiac output or function is rather inferior.^1^ For instance, phase 2, randomized, double-blind, placebo-controlled trial (PreSERVE-AMI) demonstrated the safety and bioactivity of intracoronary administration of the BM-derived autologous CD34+ cells in patients having residual LVD post-ST segment elevation MI (STEMI). Upon adjusting the ischemic time, CD34+ cell dose-dependent improvements were significant in terms of greater left ventricular ejection fraction (LVEF) change, reduced infarct size, and increased survival period. In addition, the study also indicated low mortality rates among the control (3.6%) and treatment (0%) groups and highlighted the clinical success of infusing autologous CD34 cells. This study represents the largest clinical trial that had been completed successfully for STEMI patients in USA using cell-based therapy.^58^ However, the randomized placebo-controlled, double blind-BOne marrOw transfer to enhance ST-elevation infarct regeneration (BOOST)-2 trial that had included 153 patients did not report significant improvements in LVEF between the groups when autologous nucleated BM cells were infused through the intracoronary route in patients with large STEMI.^59^ Several other limitations pertaining to available adult stem cell therapeutics include (1) challenges in analyzing the results obtained from clinical trials due to discrepancy in patients' cohort selection and variation in choice of cell population,^1^ (2) low numbers of BM-derived cells, (3) restricted replicative capacity of adult stem cells, and (4) most importantly, constraint of adult stem cells to certain lineages and their regeneration capability declining with age.^60–62^

### B. Pluripotent stem cells

Pluripotent stem cells (e.g., embryonic and induced pluripotent stem cells) are characterized by their unlimited self-renewal (stemness) and multilineage differentiation abilities and are among the most attractive cell sources for cardiac research.^63,64^ Highly purified human embryonic stem cell-derived cardiomyocytes (ESC-CMs) can be generated by exposing ESCs to activin A and bone morphogenic protein 4 (BMP4).^65^ Briefly, undifferentiated human ESCs were seeded on Matrigel-coated plates and cultured in media including a basic fibroblast growth factor (bFGF) for 6 days. For directed differentiation, ESCs were further cultured in media supplemented with human recombinant activin A for 24 h, followed by human recombinant BMP4 for another 4 days. The study hypothesized that exposing the undifferentiated ESCs to activin would lead to the mesendoderm formation and subsequently treating them with BMP4 would define the cardiac lineage. This kind of directed differentiation of ESC-CMs based on activin and BMP4 has proven efficiently cardiogenic with consistent yields of >30% and purity >82.6%. Subsequently, the differentiated cardiomyocytes were subjected to a cocktail of pro-survival factors that demonstrate enhanced survivability of cardiomyoctes *in vivo* and continue to maintain the regional and global contractile function after MI. Nevertheless, further studies are warranted for understanding mechanistic insights, and tests should be conducted for analyzing the occurrence of arrhythmic events in species with slower heart rates. Similarly, human ESC-CMs demonstrate the capability to couple electromechanically with recipient's cells, which enables synchronous contraction between the transplanted cardiomyocytes and the host tissue.^66^ In addition, the differentiated ESC-CMs engraft and promote heart regeneration when injected into the failing myocardium of a non-human primate MI model.^67^ However, the authors also reported the inevitable occurrence of concomitant ectopic arrhythmia, which was probably caused by the immature phenotype of the injected ESC-CMs. Moreover, clinical application of human ESC-CMs is hindered because of serious concerns related to genetic instability, immunogenic and tumorigenic properties, and ethical and legal debates.^68,69^ Despite these limitations, human ESCs continue to be an important laboratory tool to understand and gain insights into the pluripotency and differentiation steps in the cardiogenesis process and to generate an ideal and novel *in vitro* testing platform for cardiomyocytes.^33,52^

In order to circumvent the ethical concerns and legal debates surrounding the use of ESCs, induced pluripotent stem cells (iPSCs) have been developed by cellular programming through retroviral transduction of a defined combination of transcription factors.^70,71^ These seminal studies pave way for developing human iPSCs (hiPSCs) from adult somatic cells (e.g., skin, blood, or hair follicle) by a reprogramming process, thereby revolutionizing the field of regenerative biology.^72^ Similar to ESCs, hiPSCs are clonogenic, multipotent, and possess wide ability to differentiate into cells of any lineage.^73,74^ Although iPSCs share several common characteristics with ESCs, they have significantly different genetic profiles, microRNA patterns, and methylation signatures.^75,76^ Differentiation of hiPSCs to cardiomyocytes (hiPSC-CMs) is initiated by committing the cells to the mesodermal lineage using controlling factors such as activin A, BMP4, and/or Wnt3A.^77^ Several studies have demonstrated the use of hiPSC-CMs in different animal models (e.g., mice, rats, and pigs) to induce a cardiac regeneration post-myocardial injury.^78,79^ In addition, the cardiogenic potential of the iPSCs has been already studied with iPSCs derived from mice^80^ and humans.^81^ Despite the encouraging results of iPSCs-based therapeutics for MI, some preclinical studies have shown non-fatal ventricular arrhythmias or low survival periods in non-human primate models using hiPSC-CMs.^82^ Human pluripotent stem cell-derived cardiovascular progenitor cells (hPSC-CVPCs) were investigated in cynomolgus monkeys after MI, which showed that the transplantation of hPSC-CVPCs in acute MI monkeys can increase LV function in the short-term with immunosuppressants. However, the transplanted cells do not survive for long durations (approximately 140 days), thus excluding remuscularization. Moreover, it is challenging to cost-effectively and efficiently generate an adequate number of autologous cells within a stipulated therapeutic time period.^52^ Another important issue with iPSCs cardiogenesis is the achievement of long-term stability and its adequate integration with the host's myocardium, because of partial or incomplete differentiation of iPSCs.^52^ In addition, the pluripotency and unlimited proliferative capacity to self-replicate and differentiate may lead to teratoma formation due to either remainder of immature pluripotent cells in the differentiated lineage or impaired differentiation *in situ*.^1^ Plausible reason behind this is that the preparation of iPSCs, starting from proliferation to targeted differentiation, is time-consuming and involves a prolonged culture period. This may promote the upregulation of miRNAs that are usually seen in the cancerous state, which, in turn, enhances the probability of epigenetic and genetic deformities.^1^ Thus, as a solution, hiPSCs need to be differentiated before implantation or administration.^8,74^

Taken together, cardiomyocytes differentiated from pluripotent stem cells significantly differ from those derived from adult stem cells in terms of their structural, functional, metabolic, and molecular characteristics at the fetal stage.^14^ Although several studies have reported encouraging results, there is no gold standard on the ideal cell type to be used (or the question persists whether it may be functionally beneficial to use the combination of cells to promote both vasculogenesis and cardiomyogenesis). Moreover, following an MI event, all non-myocytes, each possessing the homeostatic function, contribute synergistically to preserve the cardiac structure and its physiological function.^83^ In Sec. III, we provide an update on the 3D bioprinting approach to fabricate the cardiac patch based on the use of either types of stem cells or a combination of cells along with different strategies to implement vascularization and augment cardiac functional properties. We also provide a comprehensive table that outlines the types of stem cells, bioinks, and 3D printing strategies adapted by different researchers globally and the subsequent significant features obtained by using the fabricated cardiac patch *in vitro* and *in vivo* (Table I).

**TABLE I.** Types of stem cells, bioinks, and 3D printing strategies adapted by researchers globally and subsequent significant features obtained using the fabricated cardiac patch *in vitro* and *in vivo*.

Cell source	Bioinks/biomaterial	Bioprinter	Number of cells	Dimensions of the patches	Significant features
hCPCs^29^	Sodium alginate, RGD-modified alginate	BioScaffolder tissue printer from SYS + ENG	30 × 10^6^ cells/ml	Final size 2 × 2 cm^2^ (after the polymerization to 1.8–1.9 cm)	Cell viability >85%
					Retention of cardiac lineage
					Upregulation of Nkx 2.5, GATA-4, Mef2c, and cTnT
					Formation of tube-like structures
hCPCs^88^	Hyaluronic-gelatin	BioScaffolder tissue printer from SYS + ENG	30 × 10^6^ cells/ml	Final size 2 × 2 cm^2^, 400 *μ*m thick	Reduced adverse remodeling
					Preservation of cardiac performance
					*In vivo* survival and engraftment of hCPCs
					Enhanced cardiac and vascular differentiation
hCPCs^91^	GelMA-hdECM	EnvisionTEC 3D-bioplotter Developer Series	1 × 10^6^ cells/ml	10 mm in diameter, 0.6 *μ*m thick, three layers, a pattern of 90° grids with 0.5 mm spacing	Cell viability >75%
					30-Fold increase in cardiospecific gene expression
					Increased angiogenic potential
					ECs tube formation
					Vascularization over 14 days *in vivo*
hiPSC-CMs^30^	Fibrin-furfuryl gelatin	Allevi 2, formerly BioBot 1	2 × 10^5^ cells/ml	Square-shape, 1 × 1 cm^2^, 500 *μ*m thick	Excellent viability, proliferation
					Expression of cTnI
Human inferior turbinate-tissue derived mesenchymal stromal cells (hTMSCs)^32^	hdECM	In-house extrusion-based 3D bioprinter	1–5 × 10^6^ cells/ml	1–10 layers, linewidth 100–200 *μ*m	>95% cell viability post-printing
					Minimal apoptotic cells
hCPCs^90^	hdECM	In-house extrusion-based 3D bioprinter	5 × 10^6^ cells/ml	Line diameters 114.2 ± 25.3–860.3 ± 67 *μ*m, up to ten layers	High cell viability and active proliferation
					Increased expression of GATA4, Nkx 2.5, MEF2C and cTnI
hMSCs, hiPSC-CMs^93^	hdECM	In-house extrusion-based 3D bioprinter	hMSCs 1 × 10^6^/ml, hiPSC-CMs 1 × 10^6^/rat	Thick 3 mm, diameter 8 mm	Amplified cardiac repair
					Improved cell retention and engraftment
					Enhanced vascular regeneration
HGF-eMSCs, BM-MSCs^97^	hdECM	3DXPrinter, (T&R Biofab)	BM-MSC 1 × 10^6^, HGF-eMSCs 1 × 10^6^, BM-MSC + HGF-eMSCs 5 × 10^5^ + 5 × 10^5^	Thick 3 mm, diameter 8 mm	Longer cell survivability
					Improved vasculogenic potential
					Enhanced vascular regeneration
					Restored cardiac function
hCPCs, hMSCs^98^	hdECM	In-house extrusion-based 3D bioprinter	hCPCs 5 × 10^6^, hMSCs 5 × 10^6^, hCPCs and MSCs 5 × 10^6^, 4 × 10^5^ cells/patch	Disk, diameter 8 mm, height 0.5 mm	Improved cell–cell interactions and differentiation
					Cellular infiltration into infarcted area
					Neomuscle and capillary formation
					Enhanced cardiac function
hiPSC-CMs, Cardiac fibroblasts (FB), HUVEC^99^	Biomaterial free	Regenova—3D bioprinter	33 000 Cells/cardiosphere, CM:FB:HUVEC (70:15:15, 70:0:30, 45:40:15 ratio)	Cardiospheres	Spontaneous beating
					Uniform electrical conductivity
					Vascularization and successful integration of patch
HUVEC, hiPSC-CMs^100^	Alginate and PEG-fibrinogen	Extrusion based with custom microfluidic printing head	HUVEC 6 × 10^6^ cells/ml, hiPSC-CM 40 × 10^6^ cells/ml	Fiber diameter 100 *μ*m, 50 *μ*m distance, ten layers-thick, perpendicular to each other (0°–90° fiber orientation), overall dimension 8 × 8 × 1 mm^3^	High orientation index of hiPSC-CMs
					Neovessel formation
					Well integration of patch
hMSCs, neonatal rat cardiomyocyte (NRCM)^95^	Gelatin	Pressure-controlled robotic dispensing system	hMSCs 5500 cells/cm^2^ NRCM 2 × 10^5^ cells/cm^2^	Microchanneled and plain hydrogel scaffold 22 × 22 mm^2^	Well defined F-actin
					Synchronized beating
hiPSC-CMs, hiPSC-smooth muscle cells (SMCs), hiPSC-ECs^96^	GelMA	Multiphoton-excited, three-dimensional printing (MPE-3DP)	Total ∼50 000 cells/patch, hiPSC-CMs:SMCs:ECs (2:1:1 ratio)	2 × 2 mm^2^, 100 *μ*m thickness	Improved cardiac function
					Reduced infarct size
					Improved vascular and arteriole density
hiPSC-CMs, hiPSC-Ecs, human neonatal dermal fibroblast (HNDF)^101^	Omenta tissue-derived dECM, gelatin	3D discovery printer (regenHU)	hiPSC-ECs, 1.5–2 × 10^7^ cell/ml, hiPSC-CMs 1 × 10^8^ cell/ml, HNDF 3 × 10^6^ cells/ml	Several mm thickness vascularized patch (thickness 2 mm), heart (height 20; diameter 14 mm)	High cell viability and contractile activity
					Elongated cardiomyocytes with massive actinin striation

## III. BIOPRINTED STEM CELL-LADEN CARDIAC PATCH

An ideal approach to regenerate a damaged myocardium is to either promote the proliferation of resident cardiomyocytes or exogenously provide cells to replace the necrotic tissue.^14^ The direct injection of cells to regeneration has shown some success in restoring myocardium function.^84^ However, most cells have been reported to die after injection. The survival time of the injected cells is short due to the lack of ECM and the supply of nutrients.^85^ In addition, it is difficult to mimic the complex properties of the native myocardium to develop cardiac tissue with constant contraction and relaxation. Therefore, special bioengineering approaches are needed to provide structures to retain the injected cells and improve regeneration.^86^

**FIG. 1.**
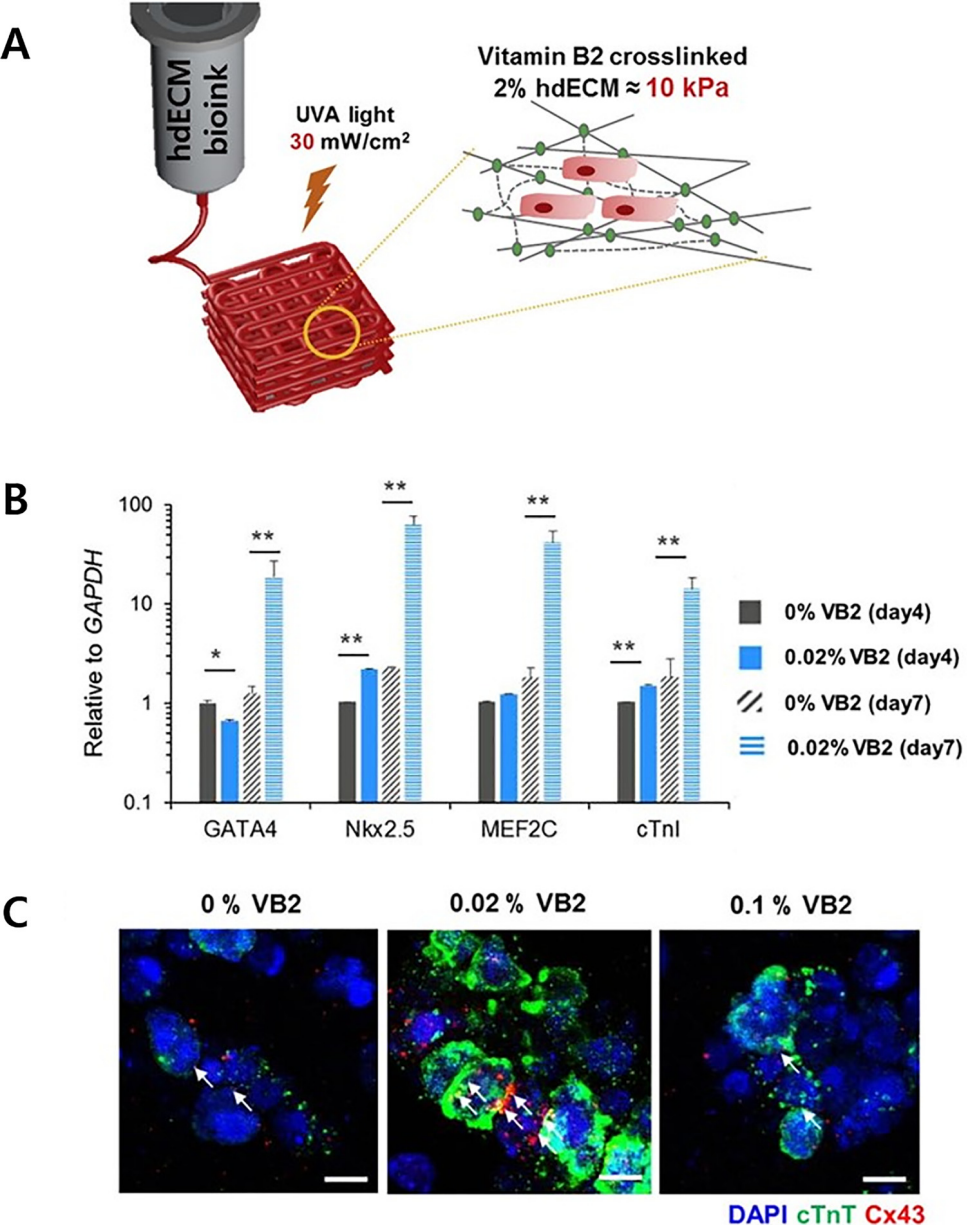
(a) Schematic representation of the two-step cross-linking procedure based on thermal and chemical cross-linking using vitamin B2 and UVA irradiation. (b) Gene expression analysis of cardiac-specific genes at days four and seven (error bars represent standard deviation, ^*^: *p* <0.05, ^**^: *p* <0.005). (c) Immunostaining images showing the expression of cardiac troponin T (cTnT, green) and connexin 43 (Cx43, red) at day seven (scale bar, 10 *μ*m; white arrows designate Cx43 expression at the cell–cell junction). [Reproduced with permission from Jang *et al.*, Acta Biomater. **33**, 88–95 (2016). Copyright 2016 Elsevier.^90^]

3D bioprinting enables the possibility to biofabricate clinically applicable, viable, and organized cardiac tissue analogs *in vitro* with the primary aim to deliver cells to the infarcted site, attract native progenitor cells for endogenous regeneration, and maintain the geometry during remodeling.^14,26^ It is strongly believed that transplantation of the bioprinted cardiac patch may facilitate significant and enhanced functional recovery post-MI.^87^ To date, researchers have creatively employed 3D bioprinting along with the use of biomimetic materials and an increasing number of cardiac cell types to recreate the native heart microenvironment. Gaetani *et al.* employed extrusion-based 3D bioprinting to develop an *in vitro* cardiac patch that has precise pore size and microstructure by using arginine-glycine-asparagine (RGD)-modified alginate^29^ and hyaluronic acid/gelatin^88^ loaded with human cardiac progenitor cells (hCPCs). These studies portrayed the applicability of bioprinting and showed better cell viability, cardiac commitment with expression of early and late cardiac markers, and *in vivo* engraftment over time. Similarly, Wang *et al.* demonstrated 3D bioprinting of cardiomyocytes loaded in a fibrin-based bioink into cardiac tissues that showed contractility with cellular organization and uniformity.^4^ Fibrin-gelatin-based 3D printed cardiac patch encompassing hiPSC-CMs could also mimic the native tissue, with respect to both cellular behavior and mechanical properties.^30,31^ In the realm of seeking novelties for therapeutic or regenerative cardiac research, it is noted that cardiomyocyte maturation significantly improves in dECM-based bioinks that represent a powerful class of biomaterials.^32^ A promising first-in-man clinical research with “VentriGel” was reported by Traverse *et al.* It is a porcine heart-derived dECM-based hydrogel that has appropriate rheology for delivery. In their study, VentriGel was applied in both early and late post-myocardial patients, from 60 days to 3 years post-MI, having LVD by transendocardial delivery, and it caused revascularization. There were no discontinued patients, side effects related to mapping and injection, or significant ventricular arrhythmias. This clinical research showed the safety and feasibility of VentriGel by percutaneous coronary intervention in the patients.^89^ In terms of printing, an initiative was taken by Pati *et al.* who demonstrated the concept and success of 3D bioprinting using specific tissue-derived dECM-based hydrogels, efficiently utilizing its thermoresponsive phenomenon to bioprint cell-laden tissue analogs with engineered porosity.^32^ The encapsulated hTMSCs in the fabricated heart dECM (hdECM)-based cardiac patch showed high cell viability post-printing and tissue-specific functionality. Taking the research further with the aim of tailoring the mechanical properties of hdECM-based bioink, Jang *et al.* reported an easy, versatile, and biocompatible two-step process to improve its printability.^90^ The physical and rheological tailoring of the bioink was done based on thermal and chemical cross-linking using vitamin B2 (0.02% VB2, also called riboflavin) and ultraviolet-A (UVA) irradiation during the bioprinting process. This two-step cross-linking procedure enhanced the stiffness of the VB2-mixed hdECM (almost 33-times) with mechanical strength comparable to that of native cardiac tissue. In addition, the bioprinted cardiac patch supported high cell viability and active proliferation of encapsulated neonatal hCPCs and demonstrated enhanced mRNA expression of the cardiac-specific genes (GATA4, Nkx 2.5, MEF2C, and cTnI) (Fig. 1). Reviewing the literature in-depth, a dECM-based 3D bioprinted cardiac patch was developed with bioinks comprising hdECM, gelatin methacrylate (GelMA), and hCPCs.^91^ The encapsulated hCPCs in the patch maintained cell viability over 75% with 30-fold surged in cardiac-specific genes compared to hCPCs in only GelMA. In addition, the conditioned media from the hdECM-based cardiac patch supported enhanced angiogenic potential (>twofold) with improvements in ECs tube formation. *In vivo* implantation in rat hearts demonstrated good retention of the cardiac patch with evidence of vascularization over 14 days. Hence, the potential therapeutic application of these patches has been suggested for pediatric patients with right ventricular heart failure or as an allogeneic reparative patch for adult cardiac dysfunction.

**FIG. 2.**
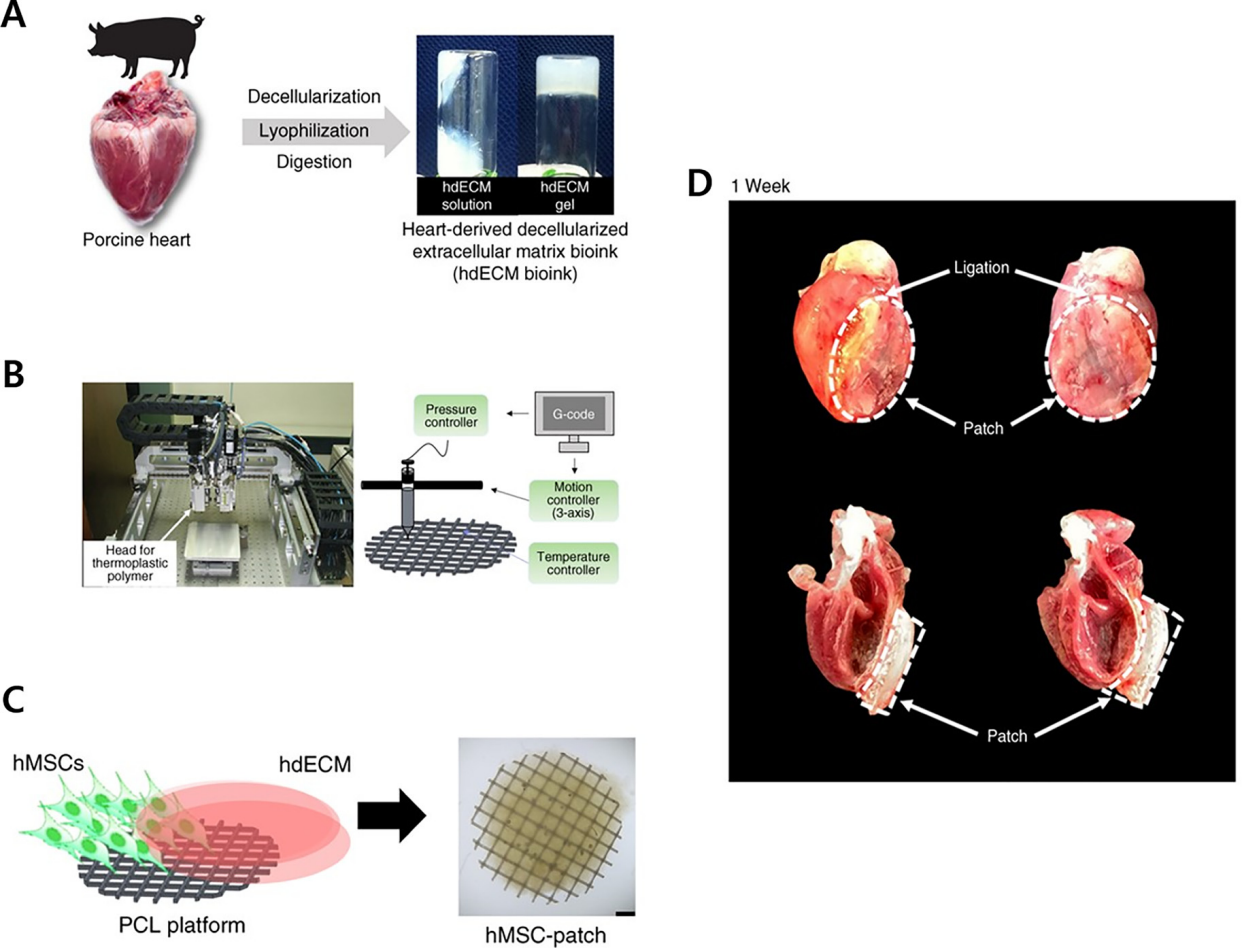
(a) Development of porcine hdECM bioink. (b) Schematic representation of fabricating the polycaprolactone (PCL) framework using an in-house extrusion-based 3D bioprinter. (c) Illustration to show the development of the hMSC-PA patch. Scale bar, 4 cm. (d) Epicardial implantation of the hMSC-PA patch in the MI heart at 1 week. [Reproduced with permission from Park *et al.*, Nat. Commun. **10**(1), 3123 (2019). Copyright 2019 Authors, licensed under a Creative Commons Attribution (CC BY) license.^93^]

**FIG. 3.**
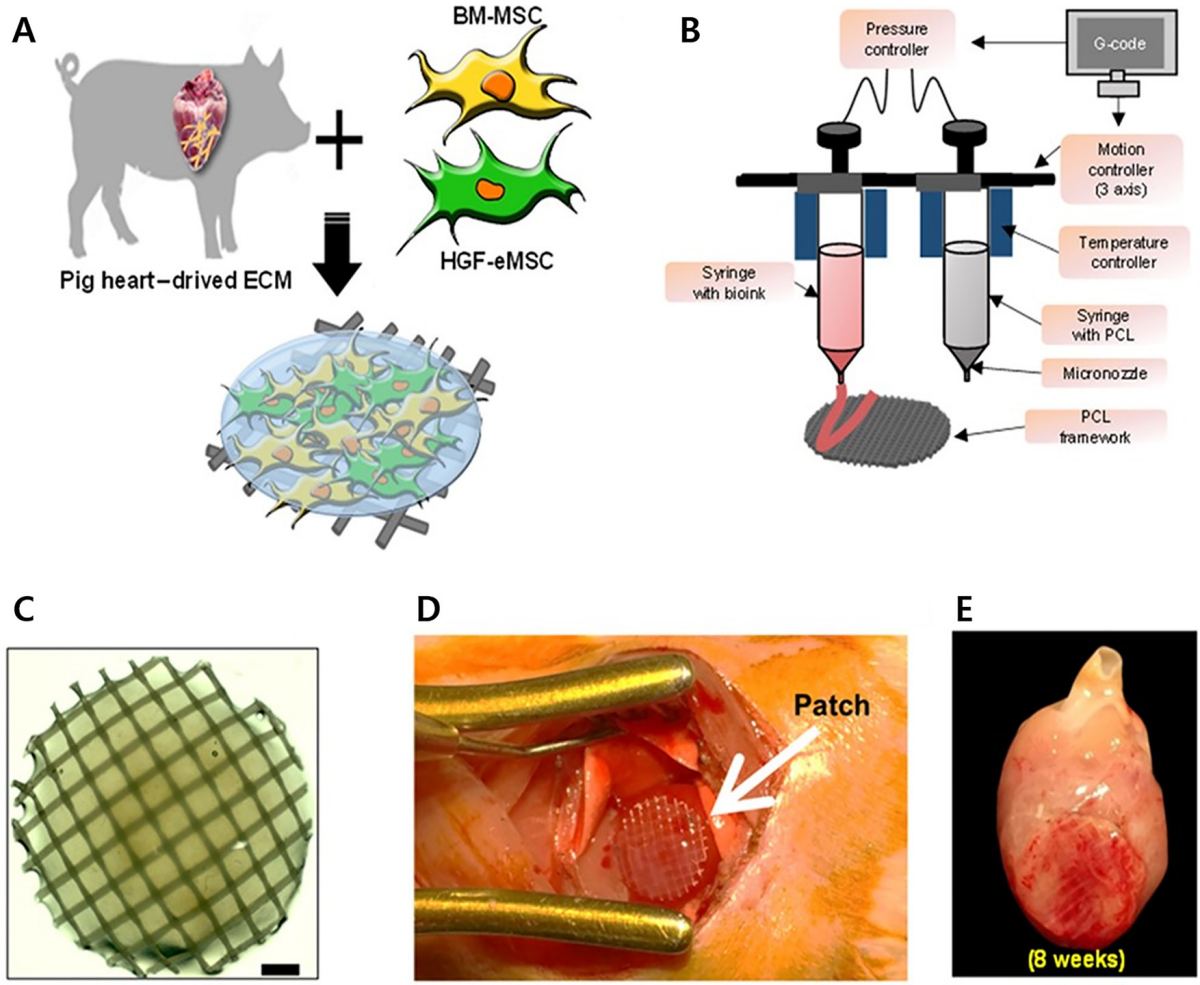
(a) Schematic representation of fabricating the BM-MSC/HGF-eMSCs patch using an hdECM bioink. (b) Illustration showing the printing process using an extrusion-based 3D bioprinter. (c) Macroscopic view of the BM-MSC/HGF-eMSC patch. Scale bar, 1 mm. (d) Epicardial implantation of the developed patch in the MI heart. (e) Implanted patch at 8 weeks. [Reproduced with permission from Park *et al.*, Sci. Adv. **6**(13), eaay6994 (2020). Copyright 2020 Authors, licensed under a Creative Commons Attribution (CC BY) license.]

Considering the efficacy and avoiding the potential undesired effects *in vivo*, the fabricated cardiac patch needs further contemplations in terms of cellular maturation, oxygen and nutrient diffusion avoiding creation of hypoxic condition, integration with host tissues, to name a few.^14,92^ Neovascularization is indeed a necessity for the stable survivability and functioning of an engraftment.^15^ Following MI aftermath, both myocardial tissues and vasculatures are equally and severely damaged; therefore, therapeutic or regenerative approaches should be planned to target both of them concurrently to achieve a successful cardiac repair.^93^ Employing a 3D bioprinting strategy to geometrically control the spatial patterning and using dual stem cell therapy or its co-culture can play an important role in promoting and synergistically improving vascularization as well as cardiac function following MI.^94–96^ In this regard, Park *et al.* demonstrated a concomitant method to examine the synergistic effects of two types of stem cells to promote cardiac repair.^93^ The reported strategy exploited the benefits of hiPSC-CMs and an hdECM-based 3D bioprinted cardiac patch loaded with human MSCs (hMSC-PA) and held the ability to substantially augment cardiac repair in a rat MI model by enhancing the retention and engraftment of transplanted cells within the myocardium. The epicardially implanted cardiac patch contributed to improved vascular regeneration due to the prolonged secretion of paracrine factors. Most importantly, significant improvement in the retention, engraftment, distribution, and maturation of intramyocardially injected hiPSC-CMs was noted, which was attributed to the secretomic milieu of the hMSC-PA cardiac patch and creating pleiotropic effects *in vivo*; collectively rejuvenating the injured myocardium and vessels post-MI (Fig. 2). However, authors reported some limitations with their complementary approach, thereby requiring considerations in further studies in terms of (1) applicability to models with advanced heart failure, (2) utilizing advanced cardiac imaging tools to ensure accurate cardiac analyses, and (3) its requirement for complicated surgical procedures for successful implantation of the cardiac patch to the heart. The research was taken further to augment the therapeutic potential of BM-MSCs.^97^ Here, Park *et al.* reported a strategy that demonstrated the *in vivo* priming of BM-MSCs through an epicardially implanted cardiac patch that was loaded with genetically engineered HGF-expressing MSCs (HGF-eMSCs).^97^ BM-MSCs encapsulated in the cardiac patch in rat MI hearts were primed through sustained release of paracrine factors (particularly HGF) by the genetically engineered HGF-eMSCs (Fig. 3). Subsequently, primed BM-MSCs in the patch survived longer, released higher amounts of beneficial paracrine factors, and conferred cardioprotection, as demonstrated by significantly high cardiomyocytes viability in the MI region. In addition, the results exhibited improved vasculogenic potential that promoted increased vascular regeneration and restored the lost cardiac functions. This remarkable study provides evidence that *in vivo* priming can be an efficient approach to maximize the therapeutic potential of BM-MSCs and enhance cardiac repair of infarcted hearts.

**FIG. 4.**
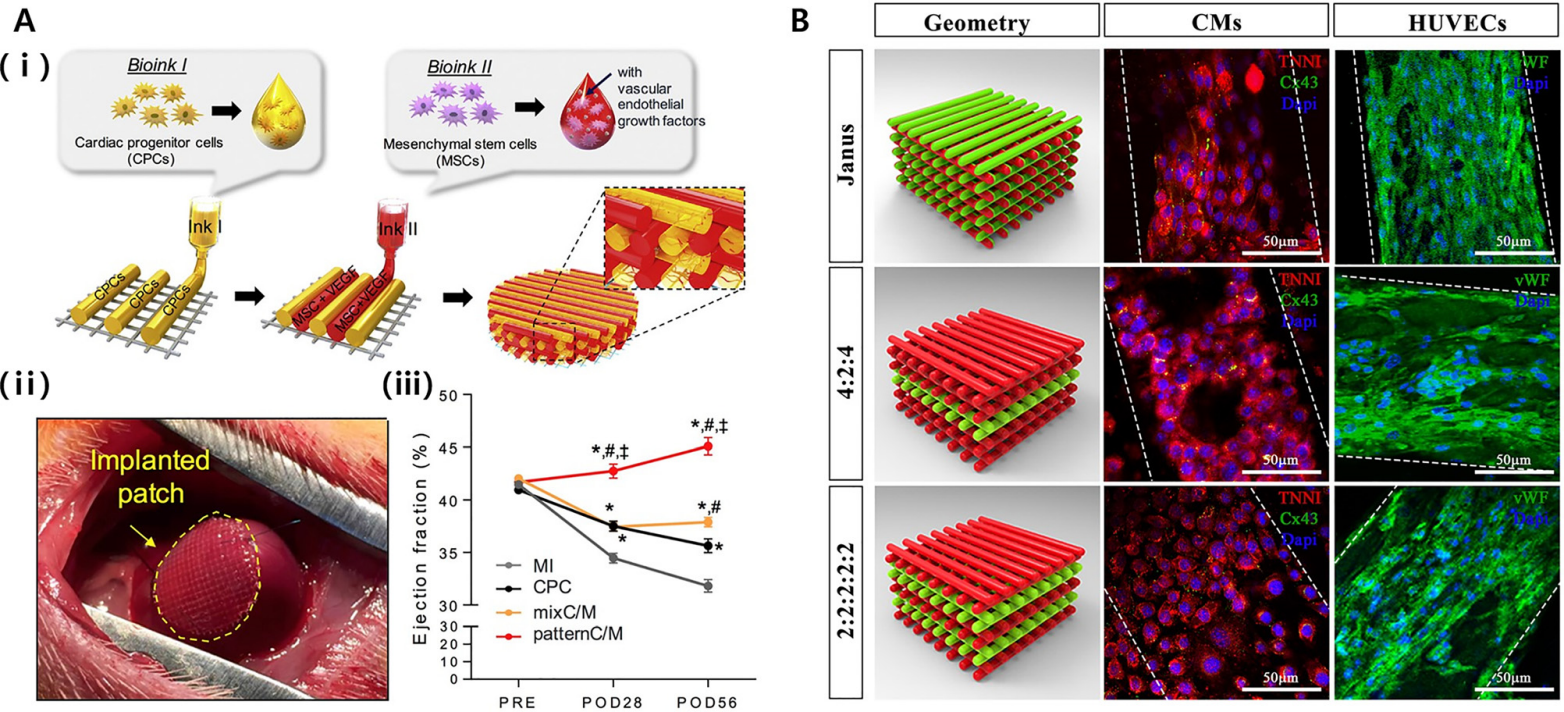
(a) (i) Schematic representation of fabricating the multicellular and multilayered pre-vascularized cardiac patch by using an hdECM bioink and the supporting PCL framework. (ii) Macroscopic view of the implanted patch. (iii) Ejection fraction values at baseline and after 4 and 8 weeks. Error bars represent (^*^p <0.05 comparison with MI; #*p* < 0.05 comparison with CPC; ǂ*p* < 0.05 comparison with mixed group C/M containing both CPC and MSCs), POD: post-operative day. [Reproduced with permission from Jang *et al.*, Biomaterials **112**, 264–274 (2017). Copyright 2017 Elsevier.^98^] (b) Janus-based 3D bioprinted cardiac tissue constructs consisting of HUVEC and hiPSC-CMs. Representative images showing the expression of cardiac troponin I (TNNI, red) and Cx43 (green) in cardiomyocytes and the von Willebrand factor (vWF, green) in HUVECs at seven days. Scale bar, 50 *μ*m. [Reproduced with permission from Maiullari *et al.*, Sci. Rep. **8**(1), 13532 (2018). Copyright 2018 Authors, licensed under a Creative Commons Attribution (CC BY) license.^100^]

**FIG. 5.**
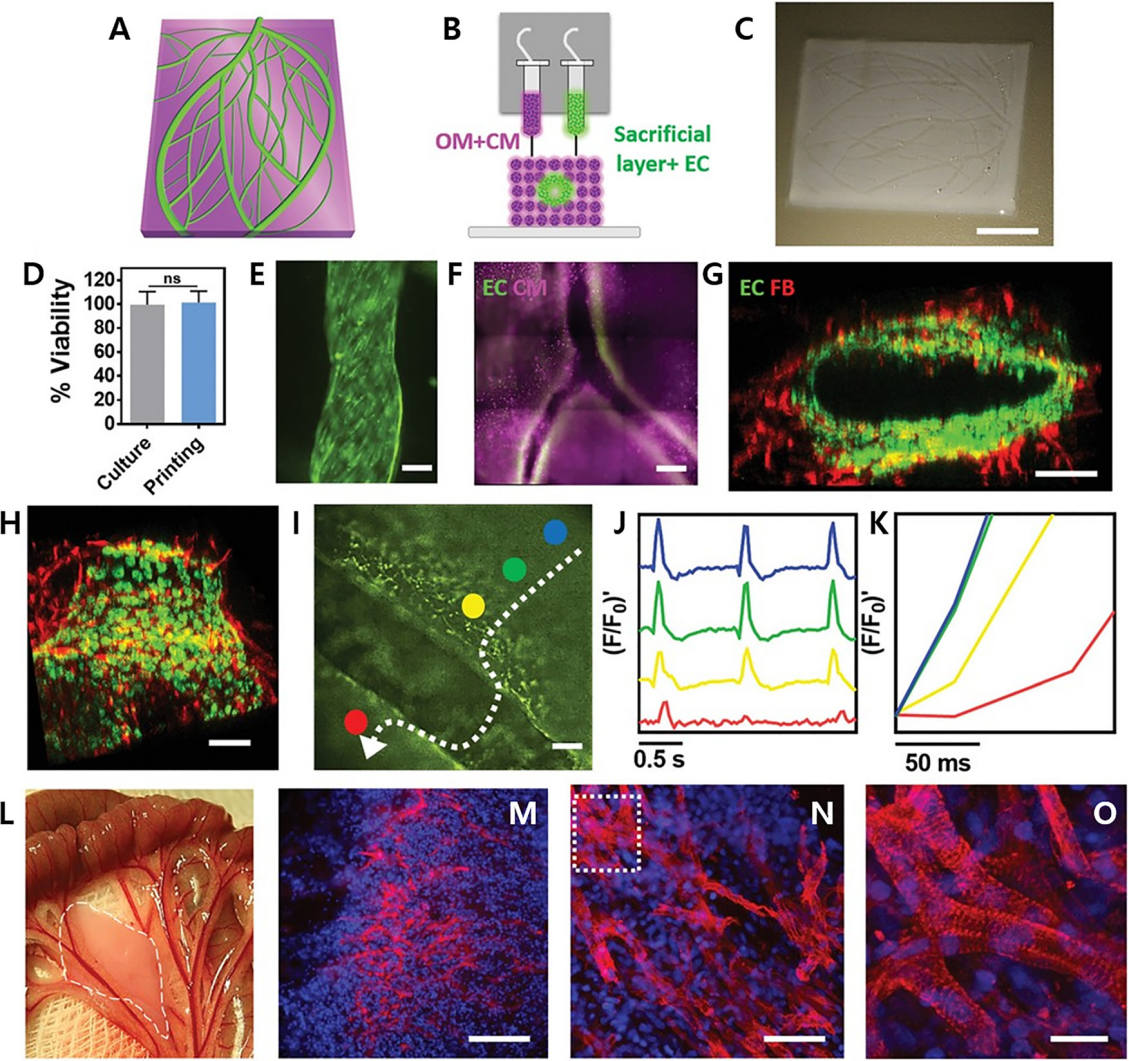
(a) A 3D model showing the cardiac patch. (b) Schematic representation of the printing concept and distinct bioinks. (c) Macroscopic view of the fabricated cardiac patch. (d) Quantitative analysis of cell viability post-printing. (e) A printed blood vessel tagged with green fluorescent protein (GFP)-expressing ECs. (f) A printed iPSCs-derived cardiac patch showing blood vessels (CD31, green) and cardiac tissue (actinin, pink). (g) and (h) Cross sections of a single lumen, illustrating the interactions of GFP and RFP (red)—expressing ECs and fibroblasts, respectively. (i) Calcium imaging of the printed vascularized cardiac patch (different colors indicate separate regions of interest and white arrow represents signal direction). (j) and (k) Quantification of calcium transients. (l) Macroscopic view of the transplanted printed patch; dashed white line indicates the patch border. (m)–(o) Immunostaining images showing the expression of sarcomeric actinin (red) and nuclei (blue). Panel (o) represents the magnified view of the marked area in (n). Scale bars: (c) = 1 cm, (e), (g), (h), (l), and (m) = 100 *μ*m, (f) = 500 *μ*m, (n) = 50 *μ*m, (o) = 25 *μ*m. [Reproduced with permission from Noor *et al.*, Adv. Sci. **6**(11), 1900344 (2019). Copyright 2019 Authors, licensed under a Creative Commons Attribution (CC BY) license.^101^]

With respect to printing, a convincing example can be found in a study that demonstrated the multicellular and multilayered 3D spatial patterning of a pre-vascularized cardiac patch by using a porcine hdECM loaded with hCPCs and MSCs and growth factors.^98^ The patch demonstrated increased cell–cell interactions and differentiation ability and improved functionality for tissue regeneration. The *in vivo* implantation of a bioprinted pre-vascularized cardiac patch to the rat MI model demonstrated cell infiltration into the infarcted area and evidenced the formation of neomuscle and capillary, in addition to an enhanced cardiac function [Fig. 4(a)]. Another interesting report illustrated the 3D bioprinting of the cardiac patch (free of biomaterial) by assembling multicellular cardiospheres constituting a mixture of hiPSC-CMs, fibroblasts (FBs), and ECs in varied ratios.^99^ The 3D bioprinted cardiac patch from each ratio displayed spontaneous beating and ventricular-like action potential waveforms, along with uniform electrical conductivity. Additionally, immunohistochemistry revealed the presence of rudimentary CD31+ blood vessels formed by ECs. Immunofluorescence data showed the expression of cardiac gap junction protein connexin-43, which was localized to cell–cell borders. Furthermore, the patch remained stably engrafted upon implantation onto rat hearts, and *in vivo* analysis showed vascularization and successful integration of the 3D bioprinted cardiac patch into the native rat myocardium. This experimental study is a good example to show that along with the 3D bioprinting technique, co-culture of different cell types may aid in improving cell maturation, neovessel formation, and cell viability. However, there are certain limitations associated with the fabricated cardiac patch, in terms of its short-term culture, concerns related to vascularization, slow conduction velocity (due to immatured hiPSC-CMs), and inferior mechanical properties that could be attributed to the fabrication of a single layer of spheroid cells. Maiullari *et al.* reported multicellular 3D bioprinting of the cardiac patch comprising of human umbilical vein ECs (HUVECs) along with hiPSC-CMs.^100^ Cells encapsulated in alginate and polyethylene glycol (PEG)-fibrinogen were extruded through a customized microfluidic printing head, which enabled precise tailoring of their 3D spatial deposition and guaranteed a high print fidelity and resolution. The Janus-based biofabricated cardiac patch contained hiPSC-CMs with a high orientation index due to varied, yet defined geometries. The formation of neovessels demonstrated well integration of the patch with recipient's vasculature upon its *in vivo* grafting [Fig. 4(b)]. This study suggested potential application of the fabricated Janus-based cardiac patch for a reconstructive therapy to re-vascularize an ischemic or damaged organ. Recently, Noor *et al.* contributed substantially to the field by reporting the fabrication of a patient-specific thick and functional perfusable vascularized cardiac patch, eliminating the need for immunosuppression treatment.^101^ The approach adopted in the study utilized patient-derived hydrogels as bioinks with combinations of hiPSC-CMs and hiPSC-ECs followed by printing the two different cell-laden bioinks into superimposed layers, where one layer contained cardiomyocytes and the other contained ECs. The bioprinted thick and vascularized cardiac patch (2–7 mm) exhibited high cellular viability and contractile activity (Fig. 5). Furthermore, the assessment of cardiac cell morphology post-transplantation revealed elongated cardiomyocytes with massive actinin striation. In addition, the authors utilized this approach to engineer small-scale human hearts having a natural anatomical architecture and demonstrated its potential for either organ replacement or drug screening in the patient-specific biochemical microenvironment. Although the bioprinted cardiac patch demonstrated an improved therapeutic efficacy, electrical stimulation is necessary for initiating cardiomyocytes' contraction and its successful synchronization with native tissue. Ideally, the electrophysiological properties of a non-human heart are significantly distinct to those from a human heart, posing a crucial limitation to most types of cardiac patch research.^102^ Several strategies have been reported to fabricate the *in vitro* tissue-engineered cardiac patch with improved electrical conduction and generating electrophysiological properties similar to those from a native heart.^103–106^ However, with respect to cardiac regeneration or its applicability in preclinical and clinical studies, the available approaches have been seen to possess limited potential to emulate physiological functions of native adult myocardium, such as (1) excitation–contraction coupling, (2) positive force–frequency relationship, and (3) efficient energy conversion, which are notably absent.^107–111^ Moreover, with iPSCs, even if gradually improved, the cells persist the major concern of maturation following cardiac induction *in vitro*. In native, cardiomyocytes are uniquely organized to perform synchronous and spontaneous beatings and contain mitochondria, densely packed sarcomeres, sarcoplasmic or endoplasmic reticulum, and transverse tubules.^110^ Although the hiPSC-based 3D bioprinted cardiac patch could serve as platforms for a patient-specific disease and physiological studies, they are currently limited by the immature cell phenotype.^107,108,110,111^ The extended culture period, hemodynamics, and mechanical and electrical stimulation are some reported ways to mature hiPSC-CMs.^112–117^ Zhang *et al.* produced endothelialized myocardium tissue based on 3D bioprinting. A hydrogel lattices scaffold with microfibers was fabricated using a low viscosity bioink developed by mixing alginate and GelMA. HUVECs are encapsulated in this bioink and printed directly. The cells move to the edge of the microfiber to form a layer of confluent endothelium. The migration and layering of HUVECs within the hydrogel lattices microfiber scaffold were made possible by the low viscosity composite bioink and facilitated by intrinsic polarization tendency and nutrient gradients. Thereafter, hiPSC-derived cardiomyocytes were cultured on a 3D anisotropic endothelial microfiber scaffold to create an aligned myocardium tissue capable of spontaneous and synchronous contraction.^118^ Zhu *et al.* developed a nanocomposite bioink, gold nanorods incorporated gelatin methacryloly (GelMA), for 3D bioprinting of functional cardiac tissue. In general, conventional bioinks are composed of polymeric biomaterials, which have low conductivity and interfere with an efficient electrical coupling between adjacent cardiac cells. To solve this problem, the gold nanorods in the nanocomposite bioinks connect the electrical resistive pore walls of the polymer, improve intercellular bonding, and promote the synchronized contraction of the 3D bioprinting structure to enhance electrical propagation and functionality in cardiac tissue.^119^ However, the maturation *in vitro* may not conform to the developmental paradigm *in vivo*. As differentiation progresses, the response of hiPSC-CMs to the induced physical stimuli declines; therefore, the electromechanical stimulation should commence during the period of cell plasticity.^110^ A worth mentioning example comes from the reported study of Ronaldson-Bouchard *et al.*, in which the early stage hiPSC-CMs that were exposed to myocardium-like physical conditioning (e.g., electrical stimulation and mechanical forces), with gradually increasing intensity (from 2 to 6 Hz in 4 weeks), promoted cells to attain the unprecedented maturation level.^110^

Altogether, these studies indicate that stem cell-laden 3D bioprinted cardiac patches have the potential to be used as a model platform to gain insights into human cardiac functions with respect to their force, pacing activity, contractile properties, and electrophysiological parameters. In addition, with more in-depth analysis and research on its applicability for further preclinical studies and clinical trials, a 3D bioprinted cardiac patch strongly represents a promising modality or resource for the upcoming future cardiac regenerative therapies.

## IV. CONCLUSION AND FUTURE ASPECTS

Stem cell-laden bioprinted cardiac patches are a promising treatment modality for cardiac repair and regeneration for patients with ischemic cardiomyopathy. Multiple types of stem cells are suggested as candidates for cardiomyocytes regeneration. Nevertheless, there is no gold standard on the specific cell type to be used, and the search is ongoing for cardiac repair. Given the complexity of the myocardial tissue that accommodates diverse cell types, its regeneration process demands multiple coordination from varied cell types with synergistic effects, along with an established ECM microenvironment. Globally, researchers have made substantial progress in developing the hiPSC-CMs for better understanding the mechanistic aspects of these cells. In addition, advances in bioengineering approaches (3D bioprinting), along with the potentially powerful autologous cell source (hiPSC-CMs), offer a new realistic option with the capacity to resuscitate injured cardiomyocytes and vasculatures and may provide new insights into the pathogenesis of CVDs. Despite the promising preclinical results, the approach is still at its infancy for subsequent clinical translation, demanding further in-depth analyses to develop as an effective and efficient strategy to achieve cardiac regeneration *in vivo*. Significant uncertainties remain, and improvements in terms of (1) rapid vascularization, (2) functional and mechanical properties of the engineered cardiac patch, (3) perfusion by host's coronary circulation, and (4) enhanced integration with host tissue are mandatory before a bioprinted cardiac patch can be considered for routine use in relevant clinical settings for treating patients with myocardial diseases. Another challenging aspect is identifying factors that drive cardiomyocytes' synchronous electrical activity, keeping in pace with that of the host tissue as it is highly desirable in order to further ameliorate the bioprinted cardiac patch. However, as the enthusiasm for cardiac regeneration charges and science continues to advance, the 3D bioprinted cardiac patch will soon become an increasingly feasible, viable, and functional option, unblocking the barriers to achieve cardiomyocytes properties. This will open new avenues for cardiac research, paving the way toward clinical translation for treating patients with CVDs.

## AUTHOR DECLARATIONS

### Conflict of Interests

The authors have no conflicts to disclose.

### Author Contributions

S.D. and H.N. contributed equally to this work.

## Data Availability

The data that support the findings of this study are available from the corresponding author upon reasonable request.

## References

[1] H. Lemcke , N. Voronina , G. Steinhoff , and R. David , Stem Cells Int. 2018, 1909346. 10.1155/2018/1909346 29535769 PMC5822776

[2] G. A. Roth , M. D. Huffman , A. E. Moran , V. Feigin , G. A. Mensah , M. Naghavi , and C. J. Murray , Circulation 132(17), 1667–1678 (2015). 10.1161/CIRCULATIONAHA.114.008720 26503749

[3] K. Thygesen , J. S. Alpert , and H. D. White , Circulation 116(22), 2634–2653 (2007). 10.1161/CIRCULATIONAHA.107.187397 17951284

[4] Z. Wang , S. J. Lee , H. J. Cheng , J. J. Yoo , and A. Atala , Acta Biomater. 70, 48–56 (2018). 10.1016/j.actbio.2018.02.007 29452273 PMC6022829

[5] M. F. Minicucci , P. S. Azevedo , B. F. Polegato , S. A. Paiva , and L. A. Zornoff , Clin. Cardiol. 34(7), 410–414 (2011). 10.1002/clc.20922 21688276 PMC6652587

[6] P. S. Jhund and J. J. V. McMurray , Circulation 118(20), 2019–2021 (2008). 10.1161/CIRCULATIONAHA.108.813493 19001032

[7] M. N. Banerjee , R. Bolli , and J. M. Hare , Circ. Res. 123(2), 266–287 (2018). 10.1161/CIRCRESAHA.118.311217 29976692 PMC8742222

[8] J. Zhang , W. Zhu , M. Radisic , and G. Vunjak-Novakovic , Circ. Res. 123(2), 244–265 (2018). 10.1161/CIRCRESAHA.118.311213 29976691 PMC7250155

[9] O. Bergmann , S. Zdunek , A. Felker , M. Salehpour , K. Alkass , S. Bernard , S. L. Sjostrom , M. Szewczykowska , T. Jackowska , C. dos Remedios , T. Malm , M. Andra , R. Jashari , J. R. Nyengaard , G. Possnert , S. Jovinge , H. Druid , and J. Frisen , Cell 161(7), 1566–1575 (2015). 10.1016/j.cell.2015.05.026 26073943

[10] Y. Y. Leong , W. H. Ng , G. M. Ellison-Hughes , and J. J. Tan , Front. Cardiovasc. Med. 4, 47 (2017). 10.3389/fcvm.2017.00047 28770214 PMC5511846

[11] A. Terzic and A. Behfar , Trends Cardiovasc. Med. 26(5), 395–404 (2016). 10.1016/j.tcm.2016.01.003 27020904 PMC4912443

[12] J. N. Tang , J. Cores , K. Huang , X. L. Cui , L. Luo , J. Y. Zhang , T. S. Li , L. Qian , and K. Cheng , Stem Cells Transl. Med. 7(4), 354–359 (2018). 10.1002/sctm.17-0196 29468830 PMC5866934

[13] R. J. Jabbour , T. J. Owen , P. Pandey , and S. E. Harding , Expert Rev. Med. Devices 17(1), 1–3 (2020). 10.1080/17434440.2020.1700793 31809201

[14] M. Mazzola and E. D. Pasquale , Front. Bioeng. Biotechnol. 8, 455 (2020). 10.3389/fbioe.2020.00455 32528940 PMC7266938

[15] D. Y. C. Cheung , B. Duan , and J. T. Butcher , *Essentials of 3D Biofabrication and Translation*, 351–370 (2015). 10.1016/B978-0-12-800972-7.00021-9

[16] M. Alonzo , S. AnilKumar , B. Roman , N. Tasnim , and B. Joddar , Transl. Res. 211, 64–83 (2019). 10.1016/j.trsl.2019.04.004 31078513 PMC6702075

[17] F. W. A. Verheugt , Neth. Heart J. 23(2), 109–110 (2015). 10.1007/s12471-014-0642-9 25605555 PMC4315795

[18] E. Yeung , T. Fukunishi , Y. Bai , D. Bedja , I. Pitaktong , G. Mattson , A. Jeyaram , C. Lui , C. S. Ong , T. Inoue , H. Matsushita , S. Abdollahi , S. M. Jay , and N. Hibino , J. Tissue Eng. Regener. Med. 13(11), 2031–2039 (2019). 10.1002/term.2954 PMC7254497 31408915

[19] C. P. Hodgkinson , A. Bareja , J. A. Gomez , and V. J. Dzau , Circ. Res. 118(1), 95–107 (2016). 10.1161/CIRCRESAHA.115.305373 26837742 PMC4874329

[20] Z. Lin and W. T. Pu , Sci. Transl. Med. 6(239), 239rv1 (2014). 10.1126/scitranslmed.3006681 PMC4280908 24898748

[21] J. Walter , L. B. Ware , and M. A. Matthay , Lancet Respir. Med. 2(12), 1016–1026 (2014). 10.1016/S2213-2600(14)70217-6 25465643

[22] P. K. Nguyen , J. W. Rhee , and J. C. Wu , JAMA Cardiol. 1(7), 831–841 (2016). 10.1001/jamacardio.2016.2225 27557438 PMC5349705

[23] F. Wang and J. J. Guan , Adv. Drug Delivery Rev. 62(7–8), 784–797 (2010). 10.1016/j.addr.2010.03.001 20214939

[24] S. Masuda , T. Shimizu , M. Yamato , and T. Okano , Adv. Drug Delivery Rev. 60(2), 277–285 (2008). 10.1016/j.addr.2007.08.031 18006178

[25] G. V. Novakovic , T. Eschenhagen , and C. Mummery , Cold Spring Harbor Perspect. Med. 4(3), a014076 (2014). 10.1101/cshperspect.a014076 PMC3935388 24591534

[26] M. Ishigami , H. Masumoto , T. Ikuno , T. Aoki , M. Kawatou , K. Minakata , T. Ikeda , R. Sakata , J. K. Yamashita , and K. Minatoya , PLoS One 13(8), e0201650 (2018). 10.1371/journal.pone.0201650 30071102 PMC6072021

[27] A. Soto-Gutierrez , J. A. Wertheim , H. C. Ott , and T. W. Gilbert , J. Clin. Invest. 122(11), 3817–3823 (2012). 10.1172/JCI61974 23114604 PMC3484436

[28] Q. Zou , B. E. Grottkau , Z. He , L. Shu , L. Yang , M. Ma , and C. Ye , Mater. Sci. Eng. C 108, 110205 (2020). 10.1016/j.msec.2019.110205 31924015

[29] R. Gaetani , P. A. Doevendans , C. H. Metz , J. Alblas , E. Messina , A. Giacomello , and J. P. Sluijter , Biomaterials 33(6), 1782–1790 (2012). 10.1016/j.biomaterials.2011.11.003 22136718

[30] S. Anil Kumar , M. Alonzo , S. C. Allen , L. Abelseth , V. Thakur , J. Akimoto , Y. Ito , S. M. Willerth , L. Suggs , M. Chattopadhyay , and B. Joddar , ACS Biomater. Sci. Eng. 5(9), 4551–4563 (2019). 10.1021/acsbiomaterials.9b00505 32258387 PMC7117097

[31] S. AnilKumar , S. C. Allen , N. Tasnim , T. Akter , S. Park , A. Kumar , M. Chattopadhyay , Y. Ito , L. J. Suggs , and B. Joddar , J. Biomed. Mater. Res. Part B 107(2), 314–323 (2019). 10.1002/jbm.b.34123 PMC6188846 29656592

[32] F. Pati , J. Jang , D. H. Ha , S. Won Kim , J. W. Rhie , J. H. Shim , D. H. Kim , and D. W. Cho , Nat. Commun. 5, 3935 (2014). 10.1038/ncomms4935 24887553 PMC4059935

[33] J. M. Hare and S. V. Chaparro , Curr. Opin. Organ Transplant. 13(5), 536–542 (2008). 10.1097/MOT.0b013e32830fdfc4 19060539 PMC3966209

[34] Y. H. Jiang , B. N. Jahagirdar , R. L. Reinhardt , R. E. Schwartz , C. D. Keene , X. R. Ortiz-Gonzalez , M. Reyes , T. Lenvik , T. Lund , M. Blackstad , J. B. Du , S. Aldrich , A. Lisberg , W. C. Low , D. A. Largaespada , and C. M. Verfaillie , Nature 418(6893), 41–49 (2002). 10.1038/nature00870 12077603

[35] A. J. Wagers and I. L. Weissman , Cell 116(5), 639–648 (2004). 10.1016/S0092-8674(04)00208-9 15006347

[36] S. Fu , Y. Zhang , Y. Li , L. Luo , Y. Zhao , and Y. Yao , Cell Death Discovery 6(1), 68 (2020). 10.1038/s41420-020-00305-y 32821437 PMC7393487

[37] S. Y. Chong , C. K. Lee , C. Huang , Y. H. Ou , C. J. Charles , A. M. Richards , Y. R. Neupane , M. V. Pavon , O. Zharkova , G. Pastorin , and J. Wang , Int. J. Mol. Sci. 20(13), 3272 (2019). 10.3390/ijms20133272 PMC6650854 31277271

[38] K. M. Danielson and S. Das , Exosomes Microvesicles 2, 4242103 (2014). 10.5772/58390 PMC4242103 25429310

[39] A. J. Boyle , S. P. Schulman , J. M. Hare , and P. Oettgen , Circulation 114(4), 339–352 (2006). 10.1161/CIRCULATIONAHA.105.590653 16864739

[40] G. A. Ramos and J. M. Hare , Cell Transplant. 16(9), 951–961 (2007). 10.3727/096368907783338208 18293894

[41] C. Gallina , V. Turinetto , and C. Giachino , Stem Cells Int. 2015, 765846. 10.1155/2015/765846 26074978 PMC4436518

[42] R. H. Lee , A. A. Pulin , M. J. Seo , D. J. Kota , J. Ylostalo , B. L. Larson , L. Semprun-Prieto , P. Delafontaine , and D. J. Prockop , Cell Stem Cell 5(1), 54–63 (2009). 10.1016/j.stem.2009.05.003 19570514 PMC4154377

[43] S. Gojo , N. Gojo , Y. Takeda , T. Mori , H. Abe , S. Kyo , J. Hata , and A. Umezawa , Exp. Cell Res. 288(1), 51–59 (2003). 10.1016/S0014-4827(03)00132-0 12878158

[44] C. Toma , M. F. Pittenger , K. S. Cahill , B. J. Byrne , and P. D. Kessler , Circulation 105(1), 93–98 (2002). 10.1161/hc0102.101442 11772882

[45] L. C. Amado , A. P. Saliaris , K. H. Schuleri , M. St John , J. S. Xie , S. Cattaneo , D. J. Durand , T. Fitton , J. Q. Kuang , G. Stewart , S. Lehrke , W. W. Baumgartner , B. J. Martin , A. W. Heldman , and J. M. Hare , Proc. Natl. Acad. Sci. U. S. A. 102(32), 11474–11479 (2005). 10.1073/pnas.0504388102 16061805 PMC1183573

[46] A. R. Williams and J. M. Hare , Circ. Res. 109(8), 923–940 (2011). 10.1161/CIRCRESAHA.111.243147 21960725 PMC3604746

[47] R. A. Rose , H. Jiang , X. Wang , S. Helke , J. N. Tsoporis , N. Gong , S. C. J. Keating , T. G. Parker , P. H. Backx , and A. Keating , Stem Cells 26(11), 2884–2892 (2008). 10.1634/stemcells.2008-0329 18687994

[48] J. M. Hare , J. E. Fishman , G. Gerstenblith , D. L. DiFede Velazquez , J. P. Zambrano , V. Y. Suncion , M. Tracy , E. Ghersin , P. V. Johnston , J. A. Brinker , E. Breton , J. Davis-Sproul , I. H. Schulman , J. Byrnes , A. M. Mendizabal , M. H. Lowery , D. Rouy , P. Altman , C. Wong Po Foo , P. Ruiz , A. Amador , J. Da Silva , I. K. McNiece , A. W. Heldman , R. George , and A. Lardo , JAMA 308(22), 2369–2379 (2012). 10.1001/jama.2012.25321 23117550 PMC4762261

[49] A. B. Mathiasen , A. A. Qayyum , E. Jorgensen , S. Helqvist , A. Fischer-Nielsen , K. F. Kofoed , M. Haack-Sorensen , A. Ekblond , and J. Kastrup , Eur. Heart J. 36(27), 1744–1753 (2015). 10.1093/eurheartj/ehv136 25926562

[50] A. Behfar , R. Crespo-Diaz , A. Terzic , and B. J. Gersh , Nat. Rev. Cardiol. 11(4), 232–246 (2014). 10.1038/nrcardio.2014.9 24594893

[51] E. C. Perin , J. T. Willerson , C. J. Pepine , T. D. Henry , S. G. Ellis , D. X. Zhao , G. V. Silva , D. Lai , J. D. Thomas , M. W. Kronenberg , A. D. Martin , R. D. Anderson , J. H. Traverse , M. S. Penn , S. Anwaruddin , A. K. Hatzopoulos , A. P. Gee , D. A. Taylor , C. R. Cogle , D. Smith , L. Westbrook , J. Chen , E. Handberg , R. E. Olson , C. Geither , S. Bowman , J. Francescon , S. Baraniuk , L. B. Piller , L. M. Simpson , C. Loghin , D. Aguilar , S. Richman , C. Zierold , J. Bettencourt , S. L. Sayre , R. W. Vojvodic , S. I. Skarlatos , D. J. Gordon , R. F. Ebert , M. Kwak , L. A. Moyé , R. D. Simari , and Cardiovascular Cell Therapy Research Network (CCTRN), JAMA 307(16), 1717–1726 (2012). 10.1001/jama.2012.418 22447880 PMC3600947

[52] J. C. Garbern and R. T. Lee , Cell Stem Cell 12(6), 689–698 (2013). 10.1016/j.stem.2013.05.008 23746978 PMC3756309

[53] Y. Shudo , S. Miyagawa , H. Ohkura , S. Fukushima , A. Saito , M. Shiozaki , N. Kawaguchi , N. Matsuura , T. Shimizu , T. Okano , A. Matsuyama , and Y. Sawa , Tissue Eng. Part A 20(3–4), 728 (2014). 10.1089/ten.tea.2012.0534 24164292 PMC3926175

[54] A. P. Beltrami , L. Barlucchi , D. Torella , M. Baker , F. Limana , S. Chimenti , H. Kasahara , M. Rota , E. Musso , K. Urbanek , A. Leri , J. Kajstura , B. Nadal-Ginard , and P. Anversa , Cell 114(6), 763–776 (2003). 10.1016/S0092-8674(03)00687-1 14505575

[55] E. Messina , L. De Angelis , G. Frati , S. Morrone , S. Chimenti , F. Fiordaliso , M. Salio , M. Battaglia , M. V. G. Latronico , M. Coletta , E. Vivarelli , L. Frati , G. Cossu , and A. Giacomello , Circ. Res. 95(9), 911–921 (2004). 10.1161/01.RES.0000147315.71699.51 15472116

[56] R. R. Smith , L. Barile , H. C. Cho , M. K. Leppo , J. M. Hare , E. Messina , A. Giacomello , M. R. Abraham , and E. Marban , Circulation 115(7), 896–908 (2007). 10.1161/CIRCULATIONAHA.106.655209 17283259

[57] M. C. Keith , X. L. Tang , Y. Tokita , Q. H. Li , S. Ghafghazi , J. M. Iv , K. U. Hong , B. Elmore , A. Amraotkar , B. L. Ganzel , K. J. Grubb , M. P. Flaherty , G. Hunt , B. Vajravelu , M. Wysoczynski , and R. Bolli , PLoS One 10(4), e0124227 (2015). 10.1371/journal.pone.0124227 25905721 PMC4408046

[58] A. A. Quyyumi , A. Vasquez , D. J. Kereiakes , M. Klapholz , G. L. Schaer , A. Abdel-Latif , S. Frohwein , T. D. Henry , R. A. Schatz , N. Dib , C. Toma , C. J. Davidson , G. W. Barsness , D. M. Shavelle , M. Cohen , J. Poole , T. Moss , P. Hyde , A. M. Kanakaraj , V. Druker , A. Chung , C. Junge , R. A. Preti , R. L. Smith , D. J. Mazzo , A. Pecora , and D. W. Losordo , Circ. Res. 120(2), 324–331 (2017). 10.1161/CIRCRESAHA.115.308165 27821724 PMC5903285

[59] K. C. Wollert , G. P. Meyer , J. Muller-Ehmsen , C. Tschope , V. Bonarjee , A. I. Larsen , A. E. May , K. Empen , E. Chorianopoulos , U. Tebbe , J. Waltenberger , H. Mahrholdt , B. Ritter , J. Pirr , D. Fischer , M. Korf-Klingebiel , L. Arseniev , H. G. Heuft , J. E. Brinchmann , D. Messinger , B. Hertenstein , A. Ganser , H. A. Katus , S. B. Felix , M. P. Gawaz , K. Dickstein , H. P. Schultheiss , D. Ladage , S. Greulich , and J. Bauersachs , Eur. Heart J. 38(39), 2936–2943 (2017). 10.1093/eurheartj/ehx188 28431003

[60] S. Golpanian , J. El-Khorazaty , A. Mendizabal , D. L. DiFede , V. Y. Suncion , V. Karantalis , J. E. Fishman , E. Ghersin , W. Balkan , and J. M. Hare , J. Am. Coll. Cardiol. 65(2), 125–132 (2015). 10.1016/j.jacc.2014.10.040 25593053 PMC4405121

[61] P. J. Kim , M. Mahmoudi , X. H. Ge , Y. Matsuura , I. Toma , S. Metzler , N. G. Kooreman , J. Ramunas , C. Holbrook , M. V. McConnell , H. Blau , P. Harnish , E. Rulifson , and P. C. Yang , Circ. Res. 116(7), e40 (2015). 10.1161/CIRCRESAHA.116.304668 25654979 PMC4380765

[62] M. R. Rosen , R. J. Myerburg , D. P. Francis , G. D. Cole , and E. Marban , J. Am. Coll. Cardiol. 64(9), 922–937 (2014). 10.1016/j.jacc.2014.06.1175 25169179 PMC4209166

[63] R. Duelen and M. Sampaolesi , EBioMedicine 16, 30–40 (2017). 10.1016/j.ebiom.2017.01.029 28169191 PMC5474503

[64] J. A. Thomson , J. Itskovitz-Eldor , S. S. Shapiro , M. A. Waknitz , J. J. Swiergiel , V. S. Marshall , and J. M. Jones , Science 282(5391), 1145–1147 (1998). 10.1126/science.282.5391.1145 9804556

[65] M. A. Laflamme , K. Y. Chen , A. V. Naumova , V. Muskheli , J. A. Fugate , S. K. Dupras , H. Reinecke , C. Xu , M. Hassanipour , S. Police , C. O'Sullivan , L. Collins , Y. Chen , E. Minami , E. A. Gill , S. Ueno , C. Yuan , J. Gold , and C. E. Murry , Nat. Biotechnol. 25(9), 1015–1024 (2007). 10.1038/nbt1327 17721512

[66] Y. Shiba , S. Fernandes , W. Z. Zhu , D. Filice , V. Muskheli , J. Kim , N. J. Palpant , J. Gantz , K. W. Moyes , H. Reinecke , B. Van Biber , T. Dardas , J. L. Mignone , A. Izawa , R. Hanna , M. Viswanathan , J. D. Gold , M. I. Kotlikoff , N. Sarvazyan , M. W. Kay , C. E. Murry , and M. A. Laflamme , Nature 489(7415), 322 (2012). 10.1038/nature11317 22864415 PMC3443324

[67] J. J. Chong , X. Yang , C. W. Don , E. Minami , Y. W. Liu , J. J. Weyers , W. M. Mahoney , B. Van Biber , S. M. Cook , N. J. Palpant , J. A. Gantz , J. A. Fugate , V. Muskheli , G. M. Gough , K. W. Vogel , C. A. Astley , C. E. Hotchkiss , A. Baldessari , L. Pabon , H. Reinecke , E. A. Gill , V. Nelson , H. P. Kiem , M. A. Laflamme , and C. E. Murry , Nature 510(7504), 273–277 (2014). 10.1038/nature13233 24776797 PMC4154594

[68] J. A. Robertson , Nat. Rev. Genet. 2(1), 74–78 (2001). 10.1038/35047594 11253076

[69] G. Blin , D. Nury , S. Stefanovic , T. Neri , O. Guillevic , B. Brinon , V. Bellamy , C. Ruecker-Martin , P. Barbry , A. Bel , P. Bruneval , C. Cowan , J. Pouly , S. Mitalipov , E. Gouadon , P. Binder , A. Hagege , M. Desnos , J. F. Renaud , P. Menasche , and M. Puceat , J. Clin. Invest. 120(4), 1125–1139 (2010). 10.1172/JCI40120 20335662 PMC2846046

[70] K. Takahashi , K. Tanabe , M. Ohnuki , M. Narita , T. Ichisaka , K. Tomoda , and S. Yamanaka , Cell 131(5), 861–872 (2007). 10.1016/j.cell.2007.11.019 18035408

[71] K. Takahashi and S. Yamanaka , Cell 126(4), 663–676 (2006). 10.1016/j.cell.2006.07.024 16904174

[72] E. Kiskinis , J. Sandoe , L. A. Williams , G. L. Boulting , R. Moccia , B. J. Wainger , S. Han , T. Peng , S. Thams , S. Mikkilineni , C. Mellin , F. T. Merkle , B. N. Davis-Dusenbery , M. Ziller , D. Oakley , J. Ichida , S. D. Costanzo , N. Atwater , M. L. Maeder , M. J. Goodwin , J. Nemesh , R. E. Handsaker , D. Paull , S. Noggle , S. A. McCarroll , J. K. Joung , C. J. Woolf , R. H. Brown , and K. Eggan , Cell Stem Cell 14(6), 873–873 (2014). 10.1016/j.stem.2014.04.005 PMC4653065 24704492

[73] J. H. Lee , J. B. Lee , Z. Shapovalova , A. Fiebig-Comyn , R. R. Mitchell , S. Laronde , E. Szabo , Y. D. Benoit , and M. Bhatia , Nat. Commun. 5, 5605 (2014). 10.1038/ncomms6605 25465724

[74] J. Zhang , Curr. Treat. Options Cardiovasc. Med. 17(8), 399 (2015) 10.1007/s11936-015-0399-5. 26122908 PMC4676725

[75] M. H. Chin , M. J. Mason , W. Xie , S. Volinia , M. Singer , C. Peterson , G. Ambartsumyan , O. Aimiuwu , L. Richter , J. Zhang , I. Khvorostov , V. Ott , M. Grunstein , N. Lavon , N. Benvenisty , C. M. Croce , A. T. Clark , T. Baxter , A. D. Pyle , M. A. Teitell , M. Pelegrini , K. Plath , and W. E. Lowry , Cell Stem Cell 5(1), 111–123 (2009). 10.1016/j.stem.2009.06.008 19570518 PMC3448781

[76] M. Gherghiceanu , L. Barad , A. Novak , I. Reiter , J. Itskovitz-Eldor , O. Binah , and L. M. Popescu , J. Cell Mol. Med. 15(11), 2539–2551 (2011). 10.1111/j.1582-4934.2011.01417.x 21883888 PMC3822963

[77] S. J. Kattman , A. D. Witty , M. Gagliardi , N. C. Dubois , M. Niapour , A. Hotta , J. Ellis , and G. Keller , Cell Stem Cell 8(2), 228–240 (2011). 10.1016/j.stem.2010.12.008 21295278

[78] T. J. Nelson , A. Martinez-Fernandez , S. Yamada , C. Perez-Terzic , Y. Ikeda , and A. Terzic , Circulation 120(5), 408–416 (2009). 10.1161/CIRCULATIONAHA.109.865154 19620500 PMC2768575

[79] L. Ye , Y. H. Chang , Q. Xiong , P. Y. Zhang , L. Y. Zhang , P. Somasundaram , M. Lepley , C. Swingen , L. P. Su , J. S. Wendel , J. Guo , A. Jang , D. Rosenbush , L. Greder , J. R. Dutton , J. H. Zhang , T. J. Kamp , D. S. Kaufman , Y. Ge , and J. Y. Zhang , Cell Stem Cell 15(6), 750–761 (2014). 10.1016/j.stem.2014.11.009 25479750 PMC4275050

[80] C. Mauritz , K. Schwanke , M. Reppel , S. Neef , K. Katsirntaki , L. S. Maier , F. Nguemo , S. Menke , M. Haustein , J. Hescheler , G. Hasenfuss , and U. Martin , Circulation 118(5), 507–517 (2008). 10.1161/CIRCULATIONAHA.108.778795 18625890

[81] J. H. Zhang , G. F. Wilson , A. G. Soerens , C. H. Koonce , J. Y. Yu , S. P. Palecek , J. A. Thomson , and T. J. Kamp , Circ. Res. 104(4), e30–e41 (2009). 10.1161/CIRCRESAHA.108.192237 19213953 PMC2741334

[82] K. Y. Zhu , Q. Wu , C. Ni , P. Zhang , Z. W. Zhong , Y. Wu , Y. C. Wang , Y. C. Xu , M. J. Kong , H. F. Cheng , Z. H. Tao , Q. Yang , H. Liang , Y. Jiang , Q. J. Li , J. Zhao , J. J. Huang , F. J. Zhang , Q. Chen , Y. Li , J. H. Chen , W. Zhu , H. Yu , J. Y. Zhang , H. T. Yang , X. Y. Hu , and J. A. Wang , Circ. Res. 122(7), 958–969 (2018). 10.1161/CIRCRESAHA.117.311578 29343525

[83] G. A. Gray , I. S. Toor , R. Castellan , M. Crisan , and M. Meloni , Curr. Opin. Physiol. 1, 46–51 (2018). 10.1016/j.cophys.2017.08.001 29876531 PMC5981027

[84] E. C. Perin , H. F. Dohmann , R. Borojevic , S. A. Silva , A. L. Sousa , C. T. Mesquita , M. I. D. Rossi , A. C. Carvalho , H. S. Dutra , H. J. F. Dohmann , G. V. Silva , L. Belém , R. Vivacqua , F. O. D. Rangel , R. Esporcatte , Y. J. Geng , W. K. Vaughn , J. A. R. Assad , E. T. Mesquita , and J. T. Willerson , Circulation 107(18), 2294–2302 (2003). 10.1161/01.CIR.0000070596.30552.8B 12707230

[85] W. H. Zimmermann , M. Didié , S. Döker , I. Melnychenko , H. Naito , C. Rogge , M. Tiburcy , and T. Eschenhagen , Cardiovasc. Res. 71(3), 419–429 (2006). 10.1016/j.cardiores.2006.03.023 16697358

[86] M. Qasim , F. Haq , M. H. Kang , and J. H. Kim , Int. J. Nanomed. 14, 1311 (2019). 10.2147/IJN.S189587 PMC6388753 30863063

[87] A. Shafiee and A. Atala , Trends Mol. Med. 22(3), 254–265 (2016). 10.1016/j.molmed.2016.01.003 26856235

[88] R. Gaetani , D. A. Feyen , V. Verhage , R. Slaats , E. Messina , K. L. Christman , A. Giacomello , P. A. Doevendans , and J. P. Sluijter , Biomaterials 61, 339–348 (2015). 10.1016/j.biomaterials.2015.05.005 26043062

[89] J. H. Traverse , T. D. Henry , N. Dib , A. N. Patel , C. Pepine , G. L. Schaer , J. A. DeQuach , A. M. Kinsey , P. Chamberlin , and K. L. Christman , JACC 4(6), 659–669 (2019). 10.1016/j.jacbts.2019.07.012 31709316 PMC6834965

[90] J. Jang , T. G. Kim , B. S. Kim , S. W. Kim , S. M. Kwon , and D. W. Cho , Acta Biomater. 33, 88–95 (2016). 10.1016/j.actbio.2016.01.013 26774760

[91] D. Bejleri , B. W. Streeter , A. L. Y. Nachlas , M. E. Brown , R. Gaetani , K. L. Christman , and M. E. Davis , Adv. Healthcare Mater. 7(23), e1800672 (2018). 10.1002/adhm.201800672 PMC6521871 30379414

[92] J. M. Lee , S. L. Sing , E. Y. S. Tan , and W. Y. Yeong , Int. J. Bioprint. 2(2), 79 (2016). 10.18063/IJB.2016.02.006

[93] S. J. Park , R. Y. Kim , B. W. Park , S. Lee , S. W. Choi , J. H. Park , J. J. Choi , S. W. Kim , J. Jang , D. W. Cho , H. M. Chung , S. H. Moon , K. Ban , and H. J. Park , Nat. Commun. 10(1), 3123 (2019). 10.1038/s41467-019-11091-2 31311935 PMC6635499

[94] J. Jang , J. Y. Park , G. Gao , and D. W. Cho , Biomaterials 156, 88–106 (2018). 10.1016/j.biomaterials.2017.11.030 29190501

[95] A. Tijore , S. A. Irvine , U. Sarig , P. Mhaisalkar , V. Baisane , and S. Venkatraman , Biofabrication 10(2), 025003 (2018). 10.1088/1758-5090/aaa15d 29235444

[96] L. Gao , M. E. Kupfer , J. P. Jung , L. Yang , P. Zhang , Y. Da Sie , Q. Tran , V. Ajeti , B. T. Freeman , V. G. Fast , P. J. Campagnola , B. M. Ogle , and J. Zhang , Circ. Res. 120(8), 1318–1325 (2017). 10.1161/CIRCRESAHA.116.310277 28069694 PMC5392171

[97] B. W. Park , S. H. Jung , S. Das , S. M. Lee , J. H. Park , H. Kim , J. W. Hwang , S. Lee , H. J. Kim , H. Y. Kim , S. Jung , D. W. Cho , J. Jang , K. Ban , and H. J. Park , Sci. Adv. 6(13), eaay6994 (2020). 10.1126/sciadv.aay6994 32284967 PMC7141892

[98] J. Jang , H. J. Park , S. W. Kim , H. Kim , J. Y. Park , S. J. Na , H. J. Kim , M. N. Park , S. H. Choi , S. H. Park , S. W. Kim , S. M. Kwon , P. J. Kim , and D. W. Cho , Biomaterials 112, 264–274 (2017). 10.1016/j.biomaterials.2016.10.026 27770630

[99] C. S. Ong , T. Fukunishi , H. Zhang , C. Y. Huang , A. Nashed , A. Blazeski , D. DiSilvestre , L. Vricella , J. Conte , L. Tung , G. F. Tomaselli , and N. Hibino , Sci. Rep. 7(1), 4566 (2017). 10.1038/s41598-017-05018-4 28676704 PMC5496874

[100] F. Maiullari , M. Costantini , M. Milan , V. Pace , M. Chirivi , S. Maiullari , A. Rainer , D. Baci , H. E. Marei , D. Seliktar , C. Gargioli , C. Bearzi , and R. Rizzi , Sci. Rep. 8(1), 13532 (2018). 10.1038/s41598-018-31848-x 30201959 PMC6131510

[101] N. Noor , A. Shapira , R. Edri , I. Gal , L. Wertheim , and T. Dvir , Adv. Sci. 6(11), 1900344 (2019). 10.1002/advs.201900344 PMC6548966 31179230

[102] M. Lux , B. Andree , T. Horvath , A. Nosko , D. Manikowski , D. Hilfiker-Kleiner , A. Haverich , and A. Hilfiker , Acta Biomater. 30, 177–187 (2016). 10.1016/j.actbio.2015.11.006 26546973

[103] T. Dvir , B. P. Timko , M. D. Brigham , S. R. Naik , S. S. Karajanagi , O. Levy , H. Jin , K. K. Parker , R. Langer , and D. S. Kohane , Nat. Nanotechnol. 6(11), 720–725 (2011). 10.1038/nnano.2011.160 21946708 PMC3208725

[104] K. Roshanbinfar , L. Vogt , B. Greber , S. Diecke , A. R. Boccaccini , T. Scheibel , and F. B. Engel , Adv. Funct. Mater. 28(42), 1803951 (2018). 10.1002/adfm.201803951

[105] B. P. Timko , T. Cohen-Karni , G. Yu , Q. Qing , B. Tian , and C. M. Lieber , Nano Lett. 9(2), 914–918 (2009). 10.1021/nl900096z 19170614 PMC2663853

[106] L. Y. Wang , J. Z. Jiang , W. X. Hua , A. Darabi , X. P. Song , C. Song , W. Zhong , M. M. Q. Xing , and X. Z. Qiu , Adv. Funct. Mater. 26(24), 4293–4305 (2016). 10.1002/adfm.201505372

[107] N. T. Feric and M. Radisic , Adv. Drug Delivery Rev. 96, 110–134 (2016). 10.1016/j.addr.2015.04.019 PMC4635107 25956564

[108] S. D. Lundy , W. Z. Zhu , M. Regnier , and M. A. Laflamme , Stem Cells Dev. 22(14), 1991–2002 (2013). 10.1089/scd.2012.0490 23461462 PMC3699903

[109] I. Mannhardt , K. Breckwoldt , D. Letuffe-Breniere , S. Schaaf , H. Schulz , C. Neuber , A. Benzin , T. Werner , A. Eder , T. Schulze , B. Klampe , T. Christ , M. N. Hirt , N. Huebner , A. Moretti , T. Eschenhagen , and A. Hansen , Stem Cell Rep. 7(1), 29–42 (2016). 10.1016/j.stemcr.2016.04.011 PMC4944531 27211213

[110] K. Ronaldson-Bouchard , S. P. Ma , K. Yeager , T. Chen , L. Song , D. Sirabella , K. Morikawa , D. Teles , M. Yazawa , and G. Vunjak-Novakovic , Nature 556(7700), 239–243 (2018). 10.1038/s41586-018-0016-3 29618819 PMC5895513

[111] X. L. Yang , L. Pabon , and C. E. Murry , Circ. Res. 114(3), 511–523 (2014). 10.1161/CIRCRESAHA.114.300558 24481842 PMC3955370

[112] C. P. Jackman , A. L. Carlson , and N. Bursac , Biomaterials 111, 66–79 (2016). 10.1016/j.biomaterials.2016.09.024 27723557 PMC5074846

[113] S. S. Nunes , J. W. Miklas , J. Liu , R. Aschar-Sobbi , Y. Xiao , B. Y. Zhang , J. H. Jiang , S. Masse , M. Gagliardi , A. Hsieh , N. Thavandiran , M. A. Laflamme , K. Nanthakumar , G. J. Gross , P. H. Backx , G. Keller , and M. Radisic , Nat. Methods 10(8), 781 (2013). 10.1038/nmeth.2524 23793239 PMC4071061

[114] M. Radisic , H. Park , H. Shing , T. Consi , F. J. Schoen , R. Langer , L. E. Freed , and G. Vunjak-Novakovic , Proc. Natl. Acad. Sci. U. S. A. 101(52), 18129–18134 (2004). 10.1073/pnas.0407817101 15604141 PMC539727

[115] I. Y. Shadrin , B. W. Allen , Y. Qian , C. P. Jackman , A. L. Carlson , M. E. Juhas , and N. Bursac , Nat. Commun. 8, 1825 (2017). 10.1038/s41467-017-01946-x 29184059 PMC5705709

[116] C. Haitao , S. Miao , T. Esworthy , X. Zhou , S. Lee , C. Liu , Z. Yu , J. P. Fisher , M. Mohiuddin , and L. G. Zhanga , Adv. Drug Delivery Rev. 132, 252–269 (2018). 10.1016/j.addr.2018.07.014 PMC6226324 30053441

[117] B. Duan , Ann. Biomed. Eng. 45(1), 195–209 (2017). 10.1007/s10439-016-1607-5 27066785

[118] Y. S. Zhang , A. Arneri , S. Bersini , S. R. Shin , K. Zhu , Z. Goli-Malekabadi , J. Aleman , C. Colosi , F. Busignani , V. Dell'Erba , C. Bishop , T. Shupe , D. Demarchi , M. Moretti , M. Rasponi , M. R. Dokmeci , A. Atala , and A. Khademhosseini , Biomaterials 110, 45–59 (2016). 10.1016/j.biomaterials.2016.09.003 27710832 PMC5198581

[119] K. Zhu , S. R. Shin , T. van Kempen , Y. Li , V. Ponraj , A. Nasajpour , S. Mandla , N. Hu , X. Liu , J. Leijten , Y. Lin , M. A. Hussain , Y. S. Zhang , A. Tamayol , and A. Khademhosseini , Adv. Funct. Mater. 27(12), 1605352 (2017). 10.1002/adfm.201605352 30319321 PMC6181228

